# Design and Validation of MEDOC, a Tool to Assess the Combined Adherence to Mediterranean and Western Dietary Patterns

**DOI:** 10.3390/nu16111745

**Published:** 2024-06-02

**Authors:** Camilla Barbero Mazzucca, Lorenza Scotti, Davide Raineri, Giuseppe Cappellano, Annalisa Chiocchetti

**Affiliations:** 1 Department of Health Sciences, Interdisciplinary Research Center of Autoimmune Diseases—IRCAD, University of Eastern Piedmont, 28100 Novara, Italy; camilla.barbero@uniupo.it (C.B.M.); davide.raineri@uniupo.it (D.R.); giuseppe.cappellano@med.uniupo.it (G.C.); 2 Center for Translational Research on Autoimmune and Allergic Disease—CAAD, Università del Piemonte Orientale, 28100 Novara, Italy; 3 Department of Translational Medicine, University of Piemonte Orientale, 28100 Novara, Italy; lorenza.scotti@uniupo.it

**Keywords:** Mediterranean diet, western diet, food frequency questionnaire, nutrition transition

## Abstract

The Mediterranean diet (MD) and Western diet (WD) are poles apart as dietary patterns. Despite the availability of epidemiological tools to estimate the adherence to MD, to date, there is a lack of combined scores. We developed MEDOC, a food frequency questionnaire (FFQ) designed to calculate a combined adherence score for both diets and validated it on 213 subjects. The test–retest reliability revealed all frequency questions falling within the acceptable range of 0.5 to 0.7 (Pearson correlation coefficient) in younger (<30 years old) subjects, while 1 question out of 39 fell below the range in older (>30 years old) participants. The reproducibility for portion size was less satisfying, with, respectively, 38.2% and 70.5% of questions falling below 0.5 (Cohen’s Kappa index) for younger and older subjects. The good correlation (R = 0.63, *p* < 0.0001 for subjects younger than 30 years and R = 0.54, *p* < 0.0001 for subjects older than 30 years, Pearson’s correlation coefficient) between the MEDOC score and the MediDietScore (MDS) confirmed the validity of the MEDOC score in identifying patients who adhere to the MD. Harnessing the capabilities of this innovative tool, we aim to broaden the existing perspective to study complex dietary patterns in nutritional epidemiology studies.

## 1. Introduction

**Figure 1:**
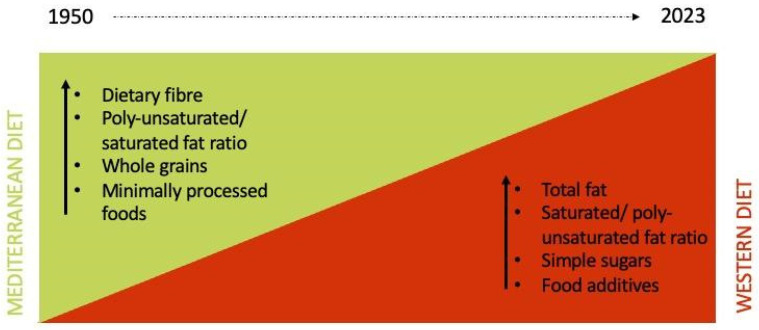
Nutrition transition from MD to WD. Arrows indicate the increase of intake of specific nutrients for each nutritional model.

The preventive potential of the Mediterranean diet (MD) [1] against the commonest chronic diseases and cancer is universally recognized [2,3,4]. However, the influence of the Western diet (WD) has dramatically grown even in the Mediterranean-basin countries, due to the transition from traditional dietary patterns to Western-like models and behaviors that has occurred over the past 100 years (Figure 1), deemed responsible for the growing incidence of obesity and chronic and autoimmune diseases [5,6].

This transition has been caused by the rise of incomes following industrialization, which led to a shift from higher energy intake and dietary consumption from rural diets, high in cereals and fiber, toward diets high in sugars, fats, and animal-source food [7]. Nutrition transition was uneven and dispersed in Europe [8], leading to complex landscapes difficult to recapitulate with traditional tools. In this context, it may be necessary to revise the most common tools for estimating adherence to MD by adapting them to the Western-like substrate [1]. Originally, MD was configured as a rural dietary pattern, characterized by the frequent and seasonal consumption of fruits, vegetables, whole grains, legumes, and olive oil and moderate consumption of animal products; however, MD has evolved and changed over the years, including also value aspects such as physical activity and conviviality [9], which, to our knowledge, are not taken into account by the MD adherence scores available to date. MD has become the reference model from which nutritional guidelines for the general population in Italy have been derived [10]. These guidelines have the goal of preventing chronic diseases and cancer, which represent the leading cause of mortality and reduced quality of life in industrialized countries [11]. The underlying evidence has been obtained estimating the adherence to MD through adherence scores derived a posteriori, i.e., on the data-driven basis, or a priori, i.e., calculating a score of adherence to a pre-defined model. After the MedDietScore (MDS) [12], one of the first a priori MD adherence scores ever proposed and used in ambulatory settings [13], many other scores have been created [14,15,16]. On the contrary, there is currently a lack of a priori scoring systems available for estimating adherence to the WD, and commonly utilized food frequency questionnaires (FFQs) fail to capture or appreciate Western-like dietary patterns like the habit of eating fast foods frequently and consuming ready and ultra-processed meals and/or refined food. Moreover, traditional cut-off-based scores impede catching those subjects in which the influence of both models are merged, as a result of the dynamism of the dietary changes that has occurred in recent years. In our previous reviews, we emphasized the significance of developing tools capable of evaluating adherence to both healthy dietary patterns like the MD and unhealthy dietary patterns like the WD in a comprehensive manner, given the contrasting effects (preventive and harmful) that they may have with respect to the development of certain diseases, such as autoimmune [1] and neurodegenerative ones [1].

**Figure 2:**
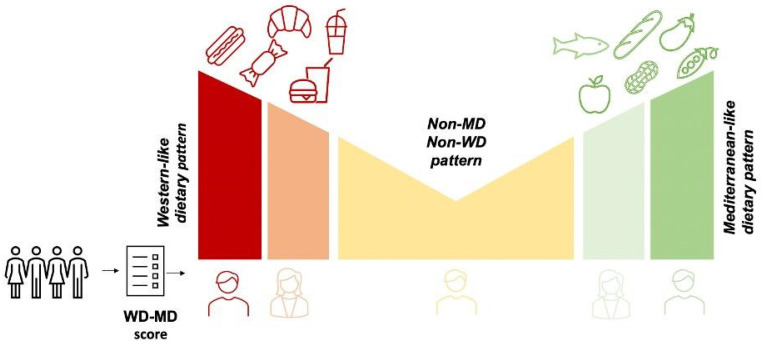
MEDOC score permits to position the subject on an adherence score running from WD (red) to MD (green), considered as opposite dietary patterns, and to appreciate subjects influenced by both dietary models (from orange to yellow to light green).

In the present study, we designed and validated MEDOC, a pioneering FFQ tailored explicitly to compute a comprehensive adherence score that encompasses both the MD and the WD in a unified manner. MEDOC allows the evaluation of the frequency of consumption of 39 items, belonging to MD and WD, accompanied by portion size and dietary behaviors. Our main aim was to develop a comprehensive instrument surpassing the limitations of traditional adherence scores. Specifically, we aimed at creating an adherence scale that spans the spectrum from WD to the MD (Figure 2), which renders it possible to evaluate varying degrees of adherence to Mediterranean patterns determined by WD influence.

## 2. Materials and Methods

### 2.1. Study Design

Before the main study phase, a pilot phase was useful to test our data collection method, instruments, and procedures to identify and address any issues or challenges. The full data collection phase involved data collection from the entire study sample or population and included tool improvements. 

Before filling the MEDOC questionnaire, all participants were asked to complete one of the latest FFQs validated to estimate the adherence to MD, that is, the MEDILITE questionnaire [16]. This was useful to test the ability of the questionnaire per se, prior to score calculation, to estimate the adherence to MD. Thus, MD adherence was calculated with both tools to assess the concordance. 

Subsequently, to test the MEDOC score’s ability to estimate adherence to MD, both MEDOC score and MDS score, i.e., the first MD adherence score ever proposed and long-term used in clinical practice, were calculated on data collected through the MEDOC FFQ. Since no reference a priori scores were available for WD, we were able to test the validity only for the MD-related part of the score. 

Having validated the score, we tested it to the study sample, in order to assess the distribution of the study sample on the double adherence scale running from WD to MD.

### 2.2. Study Sample 

Participants were recruited starting from November 2022 to February 2024 from various settings including the general population, students, and university staff, and each individual provided their signed written informed consent. All subjects were older than 18 years old. The majority of the sample consisted of females, accounting for 70% of the participants. A total of 213 subjects participated in the primary validation phase. These individuals were categorized into two age groups: the young-adults group (age ≤ 30 years) and the adults-elderly group (age > 30 years). This study was conducted according to the recommendations of the Declaration of Helsinki and was approved by the Maggiore della Carità Hospital Ethical Committee (MEDOC, 1.0, 19 May 2021). 

### 2.3. MEDOC Questionnaire Development

MEDOC is a structured semi-quantitative food frequency questionnaire (FFQ) designed to assess adherence to MD. It includes integrated questions specifically intended to calculate the adherence score for WD. This tool was designed in accordance with the guidelines outlined by Cambridge University for the creation, validation, and utilization of food frequency questionnaires [17]. It includes quantitative weekly frequency questions for the following items: fruits, vegetables, cooked vegetables, potatoes, salad, nuts, cereals, rice, legumes, refined bread, pasta, rice, fresh fish, canned fish, cured meats, white meat, red meat, cheese, seasoned cheese, eggs, legumes, pizza, milk, yogurt, biscuits, croissants, cocktails, fast foods, flatbread, fresh bread, bread substitutes, pasta, prepared meals, fried potatoes, savory snacks, pre-fried food, pastries and cakes, wine, beer, and liquors. The questionnaire incorporates qualitative inquiries, regarding portion sizes and food habits, including value aspects such as respect for seasonality and the preference to consume ready-to-eat foods or self-made meals. Participants were required to select portion sizes for each food item from a set of options depicted in images sourced from a widely referenced photographic food atlas (Atlante fotografico delle porzioni degli alimenti per adulti, Scotti&Bassani). 

### 2.4. MEDOC Score 

The purpose of the MEDOC FFQ was to construct a combined adherence score to both MD and WD. The final scoring of each participant could range between −20 and +20. A score of +20 represents complete adherence to the MD without any influence from WD, while a score of −20 identifies complete adherence to WD with no adherence to MD. Table 1 illustrates the scoring matrix used to assess the adherence score.

The questions focused on estimating adherence to MD were derived from the latest MD pyramid, updated in 2020 [18]. For each item, the frequency of consumption derived from numerical responses and was merged with the portion size (categorical questions) to define the quantity of food consumed. Recommended ranges and portion sizes for MD were used to estimate the MD adherence score, assigning a positive score to subjects consuming an amount of food falling within the recommended ranges for MD, as shown in Table 1. In some cases, 0.5 extra points were granted when the meeting of frequency requirements was accompanied by the fulfillment of matching eating behaviors reflecting the Mediterranean principles. Since MD and WD are poles apart as nutritional models, negative scores were assigned when consumption levels of items representative of MD fell outside the recommended ranges (e.g., consumption of fewer vegetables and more meat than MD requirements), and a penalty of 0.5 was conferred where both the frequency and the eating behavior did not match with the MD recommendation (e.g., eating few non-seasonal vegetables or a lot of processed non-locally produced meat). Additionally, certain specific questions, particularly those related to eating behaviors, provided informative insights indicating lower adherence to the MD and higher adherence to the unhealthy dietary model: behaviors such as having breakfast with croissants and cappuccino, dining out frequently, and eating fast food were only a few examples associated with a negative score, implying a greater adherence to WD. The WD adherence score was calculated by assigning negative points (i) to frequencies of consumption that deviated from MD requirements and (ii) to unhealthy eating behaviors. The final score was determined by summing both negative and positive points.

### 2.5. Reproducibility and Validity Assessment

To assess test–retest reliability, participants were requested to complete the questionnaire on two separate occasions; the second administration took place precisely two weeks after the first questionnaire completion. A reminder for the second round of questionnaires was sent to participants by email. Subjects who did not complete both rounds were excluded from the study (Figure S1).

For validation purposes, i.e., to assess the MEDOC questionnaire’s effectiveness in accurately gathering information to identify adherence to the MD, all participants were initially requested to complete one of the most recently validated FFQs specifically designed for estimating adherence to MD, namely the MEDILITE questionnaire [16]. It comprises nine food categories; among them, five food groups are representative of MD, and for them, 2 scoring points were assigned for the highest category of consumption, 1 for the middle, and 0 for the lowest. Conversely, for food groups not typical of MD, a value of 2 was assigned to the lowest category, 1 for the middle, and 0 for the highest. For alcohol, alcohol units were used giving 2 points to the middle consumption level, 1 point to the lowest, and 0 for the highest [16]. Consequently, adherence to the MD was computed using both tools to evaluate the agreement or concordance between them. 

Subsequently, to evaluate the MEDOC score’s capacity in estimating adherence to the MD, both the MEDOC score and MDS score, i.e., the first MD adherence score ever proposed and long-term used in clinical practice, were calculated on data collected through the MEDOC FFQ. The MDS, introduced by Panagiotakos et al., is derived from the principles of the Mediterranean dietary pattern. It assigns scores ranging from 0 to 5 to different food items based on the degree to which their consumption adheres to this dietary pattern [12]. The proposed frequency choices ranged from never to more than 32. In particular, for food items recommended for daily consumption or more than four servings per week, the scoring ranged from 0 to 5. A score of 0 denoted no consumption, while a score of 5 represented daily consumption. In contrast, for foods that deviate from this dietary pattern, like meat and meat products, the scoring was inverted. A score of 0 was assigned for nearly daily consumption, whereas a score of 5 indicated rare or no consumption. Regarding potato consumption, a score of 5 was assigned for adhering to the recommended intake of 3–4 servings per week, a score of 4 for 1–2 servings per week, and scores ranging from 3 to 0 for rare, frequent, very frequent, and daily consumption, respectively. Lastly, concerning alcohol intake, a score of 5 was given for consuming fewer than 3 wine glasses per day, a score of 0 for consuming more than 7 wine glasses per day, and scores from 4 to 1 for consuming 3, 4–5, 6, and 7 wine glasses per day. Consequently, the score spans from 0 to 55, with higher values indicating a stronger adherence to the Mediterranean dietary pattern. Since no reference a priori scores were available for WD, we were able to test the validity only for the MD-related part of the score. 

After validating the score, we applied it to the study sample, to assess the distribution of the study sample on the double adherence scale running from WD to MD.

### 2.6. Statistical Analysis

Categorical variables have been reported as absolute frequencies and percentages. Numerical variables such as mean and standard or median deviation and first and third quartiles have been reported in case the variables did not follow a Gaussian distribution. The verification of the normality of continuous variables was carried out using the Shapiro–Wilk test and through the evaluation of the quantile–quantile plot. To assess the test–retest reliability of food frequency, the Spearman’s correlation (R), the corresponding 95% confidence intervals (95%CI), and the intraclass correlation coefficient (ICC) were calculated. For both indexes, values greater than 0.8 indicate optimal reliability, values between 0.6 and 0.8 substantial reliability, between 0.4 and 0.6 moderate reliability, and values less than 0.4 poor reliability. Regarding the reproducibility assessment for portion sizes, the Cohen’s Kappa index and 95%CI were calculated. Values of the index close to the value 1 indicate strong agreement between the answers. According to the Landis and Koch scale, values greater of 0.8 are indicative of an almost perfect concordance, while values between 0.6 and 0.8 substantial agreement, between 0.4 and 0.6 moderate agreement, and below 0.4 a suboptimal agreement. Simple kappa values were reported for dichotomous variables, while weighted kappa was reported for categorical variables with more than two levels. This second index assigns different weights to the discrepancies depending on the distances between answers. To assess questionnaire validity, the correlation between the MD-related part of the MEDOC score and MEDILITE score was calculated. To evaluate the MD score validity, the correlation between the MEDOC score and MDS was calculated. Finally, descriptive statistics were also calculated for the overall double adherence scale running from the WD to MD MEDOC score and stratified according to sex, education levels, and BMI (≤median vs. >median).

## 3. Results

### 3.1. Study Sample 

The following results pertain the data collected in the main phase, including 213 participants, whose demographic characteristics are shown in Table 2. To pursue the main objective of this study, i.e., to obtain an instrument that is suitable to be used for the overall population, we categorized the study participants into two groups based on age: the young-adults and the adults-elderly population. Before starting FFQ completion, all participants were required to indicate their anthropometric measures (height, weight), from which body mass index (BMI) was calculated.

### 3.2. MEDOC FFQ Showed Good Test–Retest Reliability

The test–retest reliability of the MEDOC questionnaire demonstrated satisfactory results. 

The Pearson correlation coefficient, corresponding to 95% confidence intervals (95%CI), and intra-class correlation coefficient (ICC) were calculated to assess the test–retest reliability of food frequency intake between the two time points, separately for the young-adults (a) and the adults-elderly (b) (Table 3). The test–retest reliability revealed all questions falling within the acceptable range of 0.5 to 0.7, as proposed by Cade et al. for reproducibility trials [17], in younger (<30 years old) subjects. In older (>30 years old) subjects, 1 question out of 39 fell below the range (breakfast with croissant and cappuccino). 

The reproducibility for portion size was less satisfying, with, respectively, 38.2% and 70.5% of questions falling below 0.5 (Cohen’s Kappa index) for younger and older subjects; Table 4 shows the weighted values or simple kappa indexes and the corresponding 95%CI used to assess reproducibility of portion sizes. The reproducibility of questions regarding dietary behaviors has been described in Supplementary Table S2.

### 3.3. MEDILITE Scores Calculated on MEDOC and MEDILITE FFQ Show a Good Correlation

**Figure 3:**
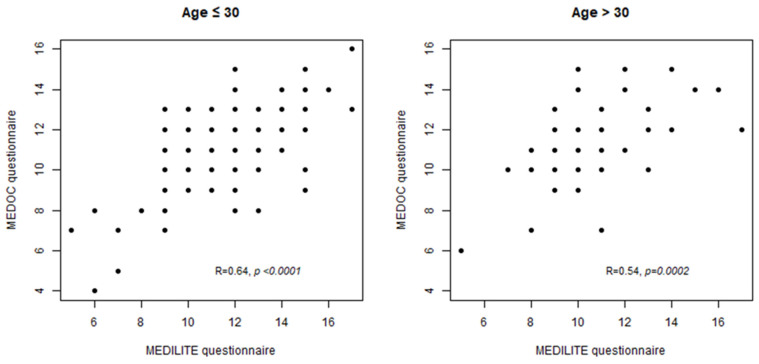
Correlation between MEDILITE score calculated, respectively, on MEDOC questionnaire and on MEDILITE questionnaire.

To assess the intrinsic capability of MEDOC FFQ to estimate the adherence to MD, the correlation between MEDILITE scores calculated on both MEDOC and MEDILITE tools was tested, showing a good concordance for both age groups (R = 0.64, *p* < 0.0001 for subjects younger than 30 years and R = 0.54 and *p* = 0.0002 for older ones). The correlation dot-plots are shown in Figure 3.

### 3.4. MD Score Estimated with MEDOC Correlates with MDS Score Used as Reference

**Figure 4:**
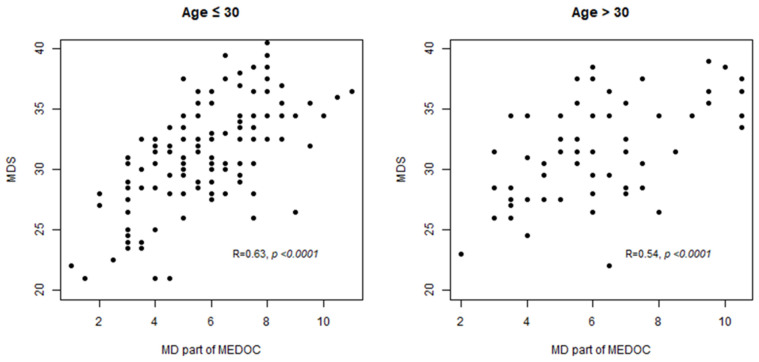
Correlation between Mediterranean diet (MD) part of MEDOC score and MD score developed by Panagiotakos et al. [12].

To validate the MEDOC score’s capability in estimating adherence to MD, we computed the correlation between the MEDOC and MDS score, used as a reference for previous questionnaire-validation studies [16]. The correlation between the two scores was satisfying in both age groups (R = 0.63, *p* < 0.0001 for subjects younger than 30 years and R = 0.54, *p* < 0.0001 for subjects older than 30 years) (Figure 4).

### 3.5. Distribution of MEDOC Score Calculated on Study Sample

**Figure 5:**
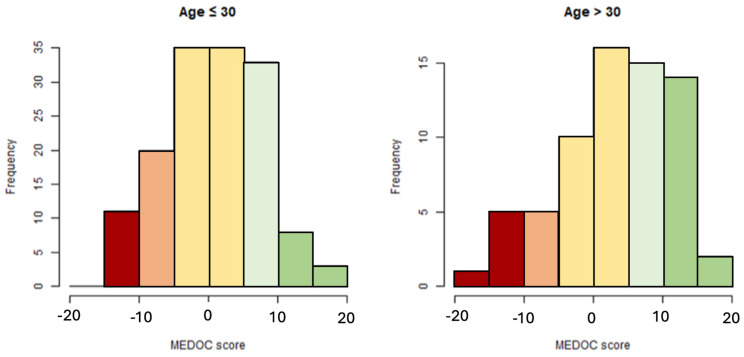
Distribution of MEDOC score in younger and older subjects. Red bars indicate subjects fully adherent to WD, orange, yellow and light green bars indicate subject that show influence of both WD and MD, while green bars indicate subjects fully adherent to MD.

The distribution of the MEDOC scores of subjects of the main study sample is shown in Figure 5, while Table 5 summarizes the statistics of MEDOC scoring (including mean, standard deviation (SD), minimum, and maximum). The mean scoring of older subjects approached more to the MD side (median 3.75, Q1–Q3 −1.00;10.50), if compared to that of younger participants (Median 1.5, Q1–Q3 −4.00;6.00), supporting the nutrition transition hypothesis.

## 4. Discussion

MD is the byword for a healthy diet [19,20] and is presently adopted as a model for nutritional guidelines due to its extensively documented preventive effects. The MD was originally defined as a dietary model typical of the Mediterranean basin in the second half of the 1990s [20]; in the last three decades, the nutrition transition has occurred overwhelmingly in industrialized countries [21]. An increasing influence of diametrically opposed dietary models, characterized by the excessive consumption of simple sugars and ultra-processed foods, defined as WD, has led to an increase in chronic diseases [12,22,23,24,25]. Parallelly, the Mediterranean model itself has been affected by socio-economic changes: thus, it is evident that the MD of the past is no longer the same as today [26,27]. In 2010, the MD was included in the intangible heritage of humanity, enriched by value aspects that configure the new conception of diet as a lifestyle [9], rather than a food frequency list. Also, the WD is characterized by unhealthy food-related behaviors, like the frequent consumption of refined and ready-to-eat meals [28,29,30]. In this progressively dynamic context, there is a growing need for new nutritional epidemiology tools that can effectively capture the complexity of dietary exposures. Thus, we developed MEDOC as a comprehensive tool able to (i) combine the effect of dietary frequencies with dietary habits and (ii) appreciate the different nuances of dietary models that coexist between healthy and unhealthy dietary patterns, i.e., MD and WD. 

MEDOC overcomes the limits of traditional dietary scores that categorize subjects as “adherent” and “non-adherent” to a specific dietary pattern, allowing us to catch those placed in between. A satisfying test–retest reproducibility of food frequency questions emerged for both considered age groups (younger or older than 30 years old). The biggest challenge was ensuring the reproducibility of the portion size inquiries. To ensure precise recognition of portion sizes, we stuck to the guidelines proposed by Ding et al. [31], inserting photographs depicting portions from a validated food atlas. Despite this, MEDOC showed a reduced ability in portion size estimation during the pilot validation phase. This challenge was particularly marked for older subjects and, while being a limitation of the study, is a commonly and frequently reported issue in nutritional research [32,33].

The good concordance between the validated MEDILITE score calculated on both MEDILITE and MEDOC tools indicated the intrinsic ability of our tool to estimate adherence to MD. Moreover, the significative correlation observed between the MD score (accounting for the “positive” part of the scoring matrix) and MDS confirmed that our tool efficiently estimates the adherence to MD pattern. A limitation of this study was the impossibility to assess the validity of the WD-related part of the score, since there was not a previously validated comparative score.

During the assessment of the study sample’s distribution on the adherence scale from WD to MD, we found support for the “nutrition transition” hypothesis. This hypothesis suggests that older subjects tend to exhibit a higher adherence to the MD model compared to younger individuals. Expanding the study sample size would be valuable to validate and further affirm our observations. The potential confirmation would imply the need to implement educational activities to raise awareness among the new generations about the importance of rediscovering healthier traditional models.

The study cohort predominantly comprised females, raising potential concerns regarding generalizability, considering that dietary habits can vary between genders. Given that MEDOC was primarily designed as an epidemiological tool to investigate the role of diet in autoimmune diseases, which are more prevalent in females, the cohort selection was aligned with this focus. Later on, it became apparent that the tool had broader applicability and could be used universally to more comprehensively characterize the influence of dietary habits on the onset/progression of various conditions. Another limitation of the study was the inability to validate the frequency questionnaire using blood biomarkers. This approach would be more reliable, even if only applicable on existing biomarkers. Nevertheless, previous validation studies tested the instrument’s validity by comparison with reference tools (e.g., MDS/MEDILITE).

## 5. Conclusions

MEDOC stands as a unique and innovative nutritional epidemiology instrument designed to explore adherence to contrasting dietary regimes, as the MD and WD are. This new tool aligns well with the modern dynamic context, where predicting adherence to intricate dietary patterns is far from straightforward. Moreover, the questionnaire was validated in two age groups allowing a broader utilization in future studies and supporting the nutrition transition hypothesis, until now only assumed but never confirmed with the use of ad hoc nutritional scores. Conventional scoring systems often struggle to address population groups where the influences of both healthy and unhealthy dietary models overlap. Consequently, we trust that MEDOC’s application in population studies will assist researchers in conducting high-quality nutritional epidemiology research, allowing a long-term evaluation of dietary exposure in clinical practices.

## Figures and Tables

**Table 1:** MEDOC scoring system.

Food Items	Cut-Off	Points	
Fruits/Vegetables	≥5 servings/day	1.5	Seasonal
	≥5 servings/day	1	Not seasonal
	<5 servings/day	−1	Seasonal
	<5 servings/day	−1.5	Not seasonal
Cereals	3–6 servings/day	1.5	Non-refined
	3–6 servings/day	1	Refined
	<3 o >6 servings/day	−1	Non-refined
	<3 o >6 servings/day	−1.5	Refined
Olive oil	Regular/Frequent consumption	1	
	Occasional consumption	−1	
Dairy	2 servings/day	1.5	Skimmed/partially skimmed
	2 servings/day	1	Full fat
	<2 o >2 servings/day	−1	Skimmed/partially skimmed
	<2 o >2 servings/day	−1.5	Full fat
Eggs	2–4 servings/week	1	
	<2 o >4 servings/week	−1	
Legumes	≥2 servings/week	1	
	<2 servings/week	−1	
Fish	≥2 servings/week	1.5	Bought by local producer
	≥2 servings/week	1	Bought at supermarket
	<2 servings/week	−1	Bought by local producer
	<2 servings/week	−1.5	Bought at supermarket
White meat	2 servings/week	1.5	Bought at supermarket
	2 servings/week	1	Bought at supermarket
	<2 o >2 servings/week	−1	Brought at supermarke
	<2 o >2 servings/week	−1.5	Bought at supermarket
Red meat	<2 servings/week	1.5	Bought by local producer
	<2 servings/week	1	Bought at supermarket
	>1 servings/week	−1	Bought by local producer
	>1 servings/week	−1.5	Bought at supermarket
Processed meat	≤1 servings/week	1	
	>1 servings/week	−1	
Sweets/cakes/pastries	≤2 servings/week	1.5	Homemade
	≤2 servings/week	1	Packed
	>2 servings/week	−1	Homemade
	>2 servings/week	−1.5	Packed
Breakfast with croissant and cappuccino	No breakfast or >1 times week	−1	
	0–1 times week	1	
Time dedicated to meals	<30 min	−1	
	≥30 min	1	
Nibbling	Yes	−1	
	No	1	
Adding spices instead of salt	No	−1	
	Yes	1	
Dining out/takeaway	≥4 times/week	−1	
	<4 times/week	1	
Bread	Weekly frequency of loaf bread > fresh bread	−1	
	Weekly frequency of loaf bread ≤ fresh bread	1	
Bread substitutes	>1 times/week	−1	
	≤1 times/week	1	
Fast food	>0 times/week	−1	
	0 times/week	1	
Salted snacks	>0 times/week	−1	
	0 times/week	1	
Sodas	>0 times/week	−1	
	0 times/week	1	
Ready-to-eat meals/Frozen food	>1 times/week	−1	
	≤1 times/week	1	

**Table 2:** Descriptive statistics of the characteristics of the study participants.

	Age ≤ 30 N = 145	Age > 30 N = 68
	Mean (SD)	Mean (SD)
Age (years)	22.91 (2.25)	56.33 (17.43)
Height (cm)	168.85 (7.78)	166.67 (9.16)
Weight (Kg)	62.36 (11.50)	64.72 (11.78)
BMI (Kg/m^2^)	21.82 (3.35)	23.18 (3.23)
	N (%)	N (%)
Sex		
Females	109 (75.17)	44 (64.71)
Males	36 (24.83)	24 (35.29)
Education level		
High school or lower	99 (68.28)	31 (45.59)
Bachelor degree	32 (22.07)	18 (26.47)
Master degree	10 (6.90)	8 (11.76)
Post-lauream	4 (2.76)	11 (16.18)
Special diet		
No	125 (87.41)	54 (85.71)
Vegetarian	6 (4.20)	1 (1.59)
Vegetarian with fish consumption	3 (2.10)	3 (4.76)
Vegan	1 (0.70)	0 (0.00)
Others	8 (5.59)	5 (7.94)
BMI classes		
Underweight (<18.5 kg/m^2^)	20 (13.89)	2 (3.13)
Healthy weight (18.5–24.9 kg/m^2^)	104 (72.22)	42 (65.63)
Overweight (25–29.9 kg/m^2^)	17 (11.91)	20 (31.25)
Obese (≥30 kg/m^2^)	3 (2.08)	0 (0.00)

**Table 3:** Number of responders, mean, standard deviation (DS), median, and first (Q1) and third (Q3) quartiles of food frequencies intake measured the first (T0) and second (T1) questionnaire’s administration. Pearson correlation coefficient, corresponding 95% confidence intervals (95%CI), and intra-class correlation coefficient (ICC) to assess test–retest reliability of food frequencies intake between the two time points among young-adults (a) and adults-elderly (b).

(a)		Age ≤ 30 Years N = 145					
		T0 Weekly Frequency		T1 Weekly Frequency			
Food Items	N	Mean (SD)	Median (Q1–Q3)	Mean (SD)	Median (Q1–Q3)	R (95%CI)	ICC
Vegetables and fruits							
Medium/large sized fruit	145	7.17 (4.77)	7 (4;10)	6.08 (4.12)	5 (3;7)	0.82 (0.75;0.86)	0.79
Small sized fruit	145	4.14 (3.2)	4 (2;6)	3.49 (3.07)	3 (1;5)	0.61 (0.50;0.71)	0.60
Dried fruit	145	2.71 (2.55)	2 (1;4)	2.64 (2.47)	2 (1;4)	0.68 (0.58;0.76)	0.68
Cooked vegetables	145	7.53 (4.26)	7 (5;10)	6.85 (4.33)	6 (4;10)	0.69 (0.60;0.77)	0.68
Raw vegetables	145	4.43 (3.61)	4 (2;6)	3.55 (2.98)	3 (2;5)	0.64 (0.54;0.73)	0.61
Salad	145	2.73 (2.79)	2 (1;4)	2.56 (2.59)	2 (1;3)	0.85 (0.80;0.89)	0.85
Potatoes	145	1.56 (1.45)	1 (1;2)	1.5 (1.26)	1 (1;2)	0.51 (0.38;0.62)	0.51
French fries	145	0.87 (1.2)	1 (0;1)	0.92 (0.98)	1 (0;1)	0.60 (0.49;0.70)	0.59
Cereals							
Breakfast cereals	145	2.12 (2.73)	1 (0;4)	2.01 (2.66)	1 (0;3)	0.78 (0.70;0.83)	0.78
Loaf bread	145	2.65 (2.98)	2 (1;3)	2.35 (2.18)	2 (1;3)	0.68 (0.59;0.76)	0.65
Fresh bread	145	4.32 (3.86)	4 (1;6)	3.96 (3.35)	3 (2;5)	0.86 (0.81;0.89)	0.84
Bread substitutes	144	3.32 (3.32)	3 (1;5)	2.61 (2.49)	2 (1;4)	0.75 (0.67;0.82)	0.70
Focaccia bread	145	1.79 (1.27)	2 (1;2)	1.86 (1.5)	2 (1;3)	0.65 (0.55;0.74)	0.64
Pizza	145	1.06 (0.57)	1 (1;1)	1.08 (0.5)	1 (1;1)	0.49 (0.36;0.61)	0.49
Rice and other cereals	145	3.22 (2.41)	3 (2;4)	2.86 (2.02)	2 (1;4)	0.72 (0.64;0.79)	0.71
Pasta	145	5.55 (2.91)	5 (3;7)	5.41 (2.94)	5 (3;7)	0.78 (0.70;0.84)	0.78
Cookies	145	3.27 (2.64)	3 (1;5)	2.92 (2.76)	3 (1;4)	0.72 (0.63;0.79)	0.72
Sweets/cakes/pastries	145	2.9 (3.04)	2 (1;4)	2.34 (2.33)	2 (1;3)	0.79 (0.72;0.85)	0.75
Dairy products							
Cow milk	145	3.02 (3.36)	2 (0;7)	3.03 (3.22)	2 (0;6)	0.85 (0.80;0.89)	0.85
Milk products	145	2.52 (2.67)	2 (0;4)	2.39 (2.63)	2 (0;4)	0.83 (0.78;0.88)	0.83
Fresh cheese	145	2.3 (1.81)	2 (1;3)	2.02 (1.54)	2 (1;3)	0.74 (0.66;0.81)	0.73
Seasoned cheese	145	1.78 (1.85)	1 (0;3)	1.51 (1.56)	1 (0;2)	0.77 (0.70;0.83)	0.75
Meat and Fish							
White meat	145	2.94 (2.23)	2 (1;4)	2.77 (2.03)	2 (2;4)	0.82 (0.75;0.86)	0.81
Red meat	145	2.18 (1.64)	2 (1;3)	2.07 (1.74)	2 (1;3)	0.74 (0.65;0.81)	0.74
Ultra-processed food	145	2.68 (2.38)	2 (1;4)	2.34 (1.98)	2 (1;3)	0.80 (0.73;0.85)	0.78
Fresh fish	145	1.31 (1.2)	1 (0;2)	1.27 (1.1)	1 (0;2)	0.79 (0.73;0.85)	0.79
Tinned fish	145	1.43 (1.34)	1 (0;2)	1.28 (1.22)	1 (0;2)	0.77 (0.70;0.83)	0.77
Eggs	144	1.81 (1.43)	2 (1;2)	1.87 (1.76)	2 (1;2)	0.58 (0.46;0.68)	0.57
Legumes	145	2.45 (2.23)	2 (1;4)	2.29 (2.22)	2 (1;3)	0.79 (0.71;0.84)	0.78
Drinks							
Soda	145	1.37 (2.24)	1 (0;2)	1.08 (1.67)	1 (0;2)	0.80 (0.73;0.85)	0.76
Wine	144	0.96 (1.11)	1 (0;1)	0.95 (1.17)	1 (0;1.5)	0.64 (0.54;0.73)	0.65
Beer	144	0.6 (1.17)	0 (0;1)	0.51 (0.78)	0 (0;1)	0.79 (0.72;0.84)	0.72
Cocktail	145	0.7 (0.72)	1 (0;1)	0.73 (0.91)	1 (0;1)	0.59 (0.47;0.68)	0.57
Spirits	145	0.26 (0.67)	0 (0;0)	0.18 (0.51)	0 (0;0)	0.62 (0.51;0.71)	0.59
Unhealthy foods							
Fast food	145	0.51 (0.71)	0 (0;1)	0.48 (0.64)	0 (0;1)	0.71 (0.62;0.79)	0.71
Salted snack	144	1.06 (1.34)	1 (0;1.5)	1.06 (1.11)	1 (0;2)	0.58 (0.47;0.68)	0.58
Frozen foods	144	0.94 (1.32)	0 (0;1)	0.86 (1.11)	1 (0;1)	0.56 (0.43;0.66)	0.55
Ready-to-eat-meals	145	1.01 (1.54)	0 (0;2)	0.89 (1.32)	0 (0;1)	0.59 (0.47;0.69)	0.58
Breakfast with croissant and cappuccino	123	1.29 (1.98)	1 (0;1)	1.27 (1.86)	1 (0;1)	0.81 (0.73;0.86)	0.82
**(b)**		**Age > 30 Years** **N = 68**					
		**T0 ** **Weekly Frequency**		**T1 ** **Weekly Frequency**			
**Food Items**	**N**	**Mean (SD)**	**Median** **(Q1–Q3)**	**Mean** **(SD)**	**Median** **(Q1–Q3)**	**R (95%CI)**	**ICC**
Vegetables and fruits							
Medium/large sized fruit	68	8.46 (5.22)	7 (5;14)	7.47 (4.65)	7 (4;11.5)	0.67 (0.51;0.78)	0.66
Small sized fruit	68	4.41 (4.6)	3 (1;6.5)	3.56 (3.64)	3 (1;5.5)	0.64 (0.48;0.76)	0.62
Dried fruit	68	3.56 (4.1)	2 (1;6)	3.21 (3.02)	3 (0;5.5)	0.65 (0.48;0.77)	0.62
Cooked vegetables	68	7.28 (4.25)	7 (4;10)	6.71 (3.8)	6 (4;9)	0.74 (0.60;0.83)	0.73
Raw vegetables	67	3.76 (3.02)	3 (1;6)	3.28 (2.81)	3 (2;4)	0.54 (0.35;0.69)	0.54
Salad	68	3.76 (2.83)	4 (2;5)	3.57 (2.66)	3 (2;5)	0.78 (0.66;0.86)	0.78
Potatoes	68	1.53 (1.23)	1 (1;2)	1.59 (1.32)	1 (1;2)	0.81 (0.71;0.88)	0.81
French fries	68	0.49 (0.86)	0 (0;1)	0.49 (0.82)	0 (0;1)	0.60 (0.42;0.73)	0.60
Cereals							
Breakfast cereals	68	1.32 (2.03)	0 (0;2)	1.34 (2.02)	0 (0;2)	0.86 (0.78;0.91)	0.86
Loaf bread	68	2.25 (3.24)	1 (0;3.5)	1.93 (2.54)	1 (0;3)	0.63 (0.47;0.76)	0.62
Fresh bread	68	5.15 (4.21)	5 (1.5;7)	5.12 (4.57)	4.5 (1.5;7)	0.85 (0.77;0.90)	0.85
Bread substitutes	68	3.76 (3.44)	3 (1;5.5)	3 (2.75)	3 (1;4)	0.70 (0.55;0.80)	0.67
Focaccia bread	68	1.06 (1.17)	1 (0;1)	0.94 (1.13)	1 (0;1)	0.52 (0.32;0.68)	0.52
Pizza	67	0.9 (0.61)	1 (1;1)	1.07 (1)	1 (1;1)	0.59 (0.40;0.72)	0.51
Rice and other cereals	68	2.49 (2.2)	2 (1;3)	2.26 (1.66)	2 (1;3)	0.59 (0.40;0.72)	0.56
Pasta	68	4.37 (3.07)	4 (2;6)	4.09 (2.39)	4 (3;5)	0.76 (0.63;0.84)	0.73
Cookies	68	3.78 (2.78)	4 (1;7)	3.75 (2.69)	4 (1.5;7)	0.70 (0.55;0.81)	0.71
Sweets/cakes/pastries	68	1.49 (2.15)	1 (0;2)	1.47 (2.26)	1 (0;2)	0.70 (0.55;0.80)	0.70
Dairy products							
Cow milk	68	3.56 (3.6)	2.5 (0;7)	3.37 (3.34)	3 (0;7)	0.80 (0.69;0.87)	0.80
Milk products	68	2.62 (2.56)	2 (0;5)	2.59 (2.58)	2 (0;4.5)	0.81 (0.71;0.88)	0.81
Fresh cheese	68	2.62 (2.31)	2 (1;3.5)	2.32 (1.86)	2 (1;3)	0.70 (0.56;0.81)	0.68
Seasoned cheese	68	2.07 (1.93)	2 (1;3)	2.21 (2.05)	2 (1;3)	0.75 (0.62;0.84)	0.75
Meat and Fish							
White meat	67	2.46 (1.92)	2 (1;3)	2.31 (1.71)	2 (1;3)	0.85 (0.77;0.91)	0.85
Red meat	67	1.73 (1.45)	1 (1;2)	1.69 (1.42)	1 (1;2)	0.68 (0.53;0.79)	0.69
Ultra-processed food	67	2.1 (2.14)	2 (1;3)	1.99 (1.73)	2 (1;3)	0.80 (0.69;0.87)	0.78
Fresh fish	68	1.46 (1.61)	1 (0;2)	1.56 (1.64)	1 (0;2)	0.72 (0.58;0.82)	0.72
Tinned fish	68	1.24 (1.11)	1 (1;2)	1.18 (1.02)	1 (1;1.5)	0.74 (0.61;0.83)	0.74
Eggs	68	1.97 (1.76)	2 (1;2)	1.84 (1.31)	2 (1;2)	0.73 (0.59;0.82)	0.70
Legumes	68	2.24 (2.13)	2 (1;3)	2.12 (1.93)	2 (1;3)	0.80 (0.69;0.87)	0.80
Drinks							
Soda	68	0.59 (1.26)	0 (0;0.5)	0.59 (1.34)	0 (0;1)	0.85 (0.77;0.91)	0.85
Wine	68	2.47 (3.42)	1 (0;4)	2.25 (3.17)	1 (0;3)	0.86 (0.78;0.91)	0.85
Beer	67	0.64 (1.08)	0 (0;1)	0.64 (0.9)	0 (0;1)	0.85 (0.76;0.90)	0.83
Cocktail	68	0.35 (0.64)	0 (0;1)	0.35 (0.69)	0 (0;0)	0.80 (0.69;0.87)	0.80
Spirits	66	0.55 (0.98)	0 (0;1)	0.53 (1.13)	0 (0;1)	0.88 (0.81;0.92)	0.87
Unhealthy foods							
Fast food	68	0.19 (0.47)	0 (0;0)	0.24 (0.67)	0 (0;0)	0.62 (0.45;0.75)	0.58
Salted snack	68	0.82 (1.18)	0 (0;1)	0.81 (1.08)	0 (0;1)	0.80 (0.69;0.87)	0.80
Frozen foods	68	0.47 (0.84)	0 (0;1)	0.5 (0.91)	0 (0;1)	0.61 (0.44;0.74)	0.61
Ready-to-eat-meals	68	1.03 (2.25)	0 (0;2)	0.68 (1.1)	0 (0;1)	0.73 (0.60;0.83)	0.57
Breakfast with croissant and cappuccino	62	1.89 (2.53)	1 (0;3)	1.08 (1.84)	0 (0;1)	0.39 (0.12;0.59)	0.36

**Table 4:** Values of weighted or simple kappa indexes and the corresponding 95%CI used to assess the reproducibility of portion sizes.

	Age ≤ 30 Years N = 145		Age > 30 Years N = 68	
Food Items	Kappa (95%CI)	N	Kappa (95%CI)	N
Vegetables and fruits				
Medium/large sized fruit	0.39 (0.25;0.53)	133	0.28 (0.06;0.50)	57
Small sized fruit	0.39 (0.25;0.54)	117	0.32 (0.07;0.56)	45
Cooked vegetables	0.48 (0.35;0.61)	131	0.42 (0.23;0.61)	58
Raw vegetables	0.36 (0.23;0.52)	116	0.35 (0.12;0.58)	47
Salad	0.51 (0.37;0.64)	103	0.47 (0.28;0.66)	58
Potatoes	0.44 (0.28;0.60)	107	0.36 (0.07;0.64)	52
French fries	0.54 (0.44;0.64)	145	0.39 (0.16;0.62)	68
Cereals				
Breakfast cereals	0.36 (0.14;0.55)	62	0.41 (0.07;0.75)	25
Loaf bread	0.68 (0.58;0.78)	134	0.62 (0.47;0.77)	63
Fresh bread	0.57 (0.44;0.69)	145	0.40 (0.21;0.58)	68
Bread substitutes	0.55 (0.44;0.66)	145	0.34 (0.16;0.52)	68
Focaccia bread	0.31 (0.2;0.42)	145	0.32 (0.14;0.49)	68
Pizza	0.37 (0.18;0.56)	145	0.52 (0.32;0.72)	68
Rice and other cereals	0.43 (0.29;0.57)	135	0.48 (0.29;0.68)	57
Pasta	0.50 (0.38;0.63)	145	0.41 (0.25;0.56)	68
Cookies	0.58 (0.49;0.67)	143	0.49 (0.36;0.61)	68
Sweets/cakes/pastries	0.60 (0.43;0.76)	145	0.48 (0.16;0.80)	68
Dairy products				
Cow milk *	0.39 (0.19;0.58)	75	0.40 (0.06;0.75)	30
Milk products	0.68 (0.52;0.84)	86	0.45 (0.21;0.69)	40
Fresh cheese	0.57 (0.46;0.68)	145	0.35 (0.19;0.50)	68
Seasoned cheese *	0.45 (0.25;0.65)	95	0.11 (−0.25;0.46)	46
Meat and Fish				
White meat	0.56 (0.45;0.68)	145	0.49 (0.33;0.65)	68
Red meat	0.62 (0.51;0.73)	145	0.58 (0.41;0.74)	68
Ultra-processed food	0.53 (0.38;0.68)	113	0.10 (−0.09;0.42)	48
Fresh fish	0.34 (0.17;0.50)	91	0.38 (0.15;0.61)	42
Tinned fish	0.52 (0.37;0.66)	93	0.42 (0.19;0.65)	46
Eggs	0.89 (0.81;0.96)	116	0.70 (0.49;0.92)	59
Legumes	0.51 (0.37;0.64)	108	0.51 (0.30;0.72)	51
Drinks				
Soda	0.66 (0.55;0.77)	145	0.59 (0.41;0.77)	68
Wine	0.68 (0.6;0.75)	141	0.83 (0.75;0.91)	64
Beer	0.66 (0.56;0.76)	139	0.62 (0.37;0.86)	67
Cocktail	0.59 (0.49;0.68)	139	0.76 (0.67;0.85)	66
Unhealthy foods				
Fast food	−0.17 (−0.33;−0.02)	54	0.85 (0.570;1.00)	7
Salted snack	0.63 (0.44;0.82)	65	0.38 (0.03;0.72)	25

* simple Kappa.

**Table 5:** Summary statistics of MEDOC score (mean and standard deviation, DS, minimum and maximum) overall and by sex, education level, and BMI.

	MEDOC Score			
	Age ≤ 30 N = 120		Age > 30 N = 53	
	Median (Q1–Q3)	Min;Max	Median (Q1–Q3)	Min;Max
All subjects	1.50 (−4.00;6.00)	−14.5;19.0	3.75 (−1.00;10.00)	−16.0;16.0
Sex				
Females	1.00 (−4.00;5.50)	−14.5;19.0	5.25 (−0.50;10.75)	−16.0;16.0
Males	1.50 (−3.50;6.75)	−11;10.5	1.75 (−3.25;6.75)	−12.5;13.5
Education level				
High school or lower	1.00 (−4.50;6.50)	−14.5;19.0	6.00(1.00;10.00)	−10.5;16.0
Bachelor degree	1.25 (−2.50;4.75)	−10.5;10.5	0.50 (−4.50;2.50)	−12.5;16.0
Master degree	0.75 (−5.50;6.50)	−7.5;12.0	13.50 (10.25;14.75)	−16.0;16.0
Post-lauream	2.25 (−4.75;6.00)	−11.0;19.0	3.50 (−7.50;6.50)	−11.5;11.0
BMI				
≤median *	1.50 (−4.50;6.50)	−14.5;14.0	3.50 (−0.75;10.50)	−16.0;16.0
>median	0.50 (−3.50;5.76)	−11.0;19.0	4.25 (−2.00;9.00)	−12.5;16.0

* The medians are 20.98 for subjects aged ≤30 years and 23.36 for subjects aged >30 years.

## Data Availability

The original contributions presented in the study are included in the article, further inquiries can be directed to the corresponding author.

## Supplementary Materials

The following supporting information can be downloaded at: https://www.mdpi.com/article/10.3390/nu16111745/s1, Figure S1: Flowchart of the inclusion criteria of study participants. Table S1: Kappa correlation coefficients (weighted and non-weighted) and corresponding 95% confidence intervals (95%CI) to assess test–retest reliability of dietary behaviors between the two time points in the pilot validation phase.

## References

[1] Mazzucca C.B., Raineri D., Cappellano G., Chiocchetti A. (2021). How to Tackle the Relationship between Autoimmune Diseases and Diet: Well Begun Is Half-Done. Nutrients.

[2] Martínez-González M.A., Salas-Salvadó J., Estruch R., Corella D., Fitó M., Ros E. (2015). Benefits of the Mediterranean Diet: Insights From the PREDIMED Study. Prog. Cardiovasc. Dis..

[3] Esposito K., Maiorino M.I., Ceriello A., Giugliano D. (2010). Prevention and Control of Type 2 Diabetes by Mediterranean Diet: A Systematic Review. Diabetes Res. Clin. Pract..

[4] Schwingshackl L., Schwedhelm C., Galbete C., Hoffmann G. (2017). Adherence to Mediterranean Diet and Risk of Cancer: An Updated Systematic Review and Meta-Analysis. Nutrients.

[5] Jaacks L.M., Vandevijvere S., Pan A., McGowan C.J., Wallace C., Imamura F., Mozaffarian D., Swinburn B., Ezzati M. (2019). The Obesity Transition: Stages of the Global Epidemic. Lancet Diabetes Endocrinol..

[6] Popkin B.M. (2012). Nutrition Transition, Diet Change, and Its Implications. Encycl. Human Nutr..

[7] Grigg D. (1995). The Nutritional Transition in Western Europe. J. Hist. Geogr..

[8] Scali J., Richard A., Gerber M. (2001). Diet Profiles in a Population Sample from Mediterranean Southern France. Public Health Nutr..

[9] Willett W.C., Sacks F., Trichopoulou A., Drescher G., Ferro-Luzzi A., Helsing E., Trichopoulos D. (1995). Mediterranean Diet Pyramid: A Cultural Model for Healthy Eating. Am. J. Clin. Nutr..

[10] Bach-Faig A., Berry E.M., Lairon D., Reguant J., Trichopoulou A., Dernini S., Medina F.X., Battino M., Belahsen R., Miranda G. (2011). Mediterranean Diet Pyramid Today. Science and Cultural Updates. Public Health Nutr..

[11] Romagnolo D.F., Selmin O.I. (2017). Mediterranean Diet and Prevention of Chronic Diseases. Nutr. Today.

[12] Panagiotakos D.B., Pitsavos C., Stefanadis C. (2006). Dietary Patterns: A Mediterranean Diet Score and Its Relation to Clinical and Biological Markers of Cardiovascular Disease Risk. Nutr. Metab. Cardiovasc. Dis..

[13] De Matteis C., Crudele L., Battaglia S., Loconte T., Rotondo A., Ferrulli R., Gadaleta R.M., Piazzolla G., Suppressa P., Sabbà C. (2023). Identification of a Novel Score for Adherence to the Mediterranean Diet That Is Inversely Associated with Visceral Adiposity and Cardiovascular Risk: The Chrono Med Diet Score (CMDS). Nutrients.

[14] Serra-Majem L., Ribas L., Ngo J., Ortega R.M., García A., Pérez-Rodrigo C., Aranceta J. (2004). Food, Youth and the Mediterranean Diet in Spain. Development of KIDMED, Mediterranean Diet Quality Index in Children and Adolescents. Public Health Nutr..

[15] Schröder H., Fitó M., Estruch R., Martínez-González M.A., Corella D., Salas-Salvadó J., Lamuela-Raventós R., Ros E., Salaverría I., Fiol M. (2011). A Short Screener Is Valid for Assessing Mediterranean Diet Adherence among Older Spanish Men and Women. J. Nutr..

[16] Sofi F., Dinu M., Pagliai G., Marcucci R., Casini A. (2017). Validation of a Literature-Based Adherence Score to Mediterranean Diet: The MEDI-LITE Score. Int. J. Food Sci. Nutr..

[17] Cade J., Thompson R., Burley V., Warm D. (2002). Development, Validation and Utilisation of Food-Frequency Questionnaires—A Review. Public Health Nutr..

[18] Serra-Majem L., Tomaino L., Dernini S., Berry E.M., Lairon D., de la Cruz J.N., Bach-Faig A., Donini L.M., Medina F.X., Belahsen R. (2020). Updating the Mediterranean Diet Pyramid towards Sustainability: Focus on Environmental Concerns. Int. J. Environ. Res. Public Health.

[19] Ventriglio A., Sancassiani F., Contu M.P., Latorre M., Di Slavatore M., Fornaro M., Bhugra D. (2020). Mediterranean Diet and Its Benefits on Health and Mental Health: A Literature Review. Clin. Pract. Epidemiol. Ment. Health.

[20] Keys A., Keys M. (1975). How to Eat Well and Stay Well the Mediterranean Way.

[21] Schmidhuber J., Shetty P. (2005). The Nutrition Transition to 2030. Why Developing Countries Are Likely to Bear the Major Burden. Acta Agric. Scand. Sect. C.

[22] Martínez-González M.A., Sánchez-Villegas A. (2004). The Emerging Role of Mediterranean Diets in Cardiovascular Epidemiology: Monounsaturated Fats, Olive Oil, Red Wine or the Whole Pattern?. Eur. J. Epidemiol..

[23] Odermatt A. (2011). The Western-Style Diet: A Major Risk Factor for Impaired Kidney Function and Chronic Kidney Disease. Am. J. Physiol. Ren. Physiol..

[24] Oikonomou E., Psaltopoulou T., Georgiopoulos G., Siasos G., Kokkou E., Antonopoulos A., Vogiatzi G., Tsalamandris S., Gennimata V., Papanikolaou A. (2018). Western Dietary Pattern Is Associated with Severe Coronary Artery Disease. Angiology.

[25] Zinöcker M.K., Lindseth I.A. (2018). The Western Diet-Microbiome-Host Interaction and Its Role in Metabolic Disease. Nutrients.

[26] Villani A., Sultana J., Doecke J., Mantzioris E. (2019). Differences in the Interpretation of a Modernized Mediterranean Diet Prescribed in Intervention Studies for the Management of Type 2 Diabetes: How Closely Does This Align with a Traditional Mediterranean Diet?. Eur. J. Nutr..

[27] Radd-Vagenas S., Kouris-Blazos A., Singh M.F., Flood V.M. (2017). Evolution of Mediterranean Diets and Cuisine: Concepts and Definitions. Asia Pac. J. Clin. Nutr..

[28] Cohen D.A., Story M. (2014). Mitigating the Health Risks of Dining Out: The Need for Standardized Portion Sizes in Restaurants. Am. J. Public Health.

[29] Elizabeth L., Machado P., Zinöcker M., Baker P., Lawrence M. (2020). Ultra-Processed Foods and Health Outcomes: A Narrative Review. Nutrients.

[30] Maldonado-Pereira L., Barnaba C., de Los Campos G., Medina-Meza I.G. (2022). Evaluation of the Nutritional Quality of Ultra-Processed Foods (Ready to Eat + Fast Food): Fatty Acids, Sugar, and Sodium. J. Food Sci..

[31] Ding Y., Yang Y., Li F., Shao Y., Sun Z., Zhong C., Fan P., Li Z., Zhang M., Li X. (2021). Development and Validation of a Photographic Atlas of Food Portions for Accurate Quantification of Dietary Intakes in China. J. Human Nutr. Diet..

[32] Faggiano F., Vineis P., Cravanzola D., Pisani P., Xompero G., Riboli E., Kaaks R. (1992). Validation of a Method for the Estimation of Food Portion Size. Epidemiology.

[33] Hunter D.J., Sampson L., Stampfer M.J., Colditz G.A., Rosner B., Willett W.C. (1988). Variability in Portion Sizes of Commonly Consumed Foods among a Population of Women in the United States. Am. J. Epidemiol..

