# Supplementary material for: Design and Validation of MEDOC, a Tool to Assess the Combined Adherence to Mediterranean and Western Dietary Patterns
Source: Nutrients. 2024 Jun 2;16(11):1745. doi: 10.3390/nu16111745 (PMC11175000; doi:10.3390/nu16111745)
Supplement: Supplementary file 1 [file nutrients-16-01745-s001.zip › nutrients-3027285-supplementary.pdf]

SUPPLEMENTARY MATERIAL

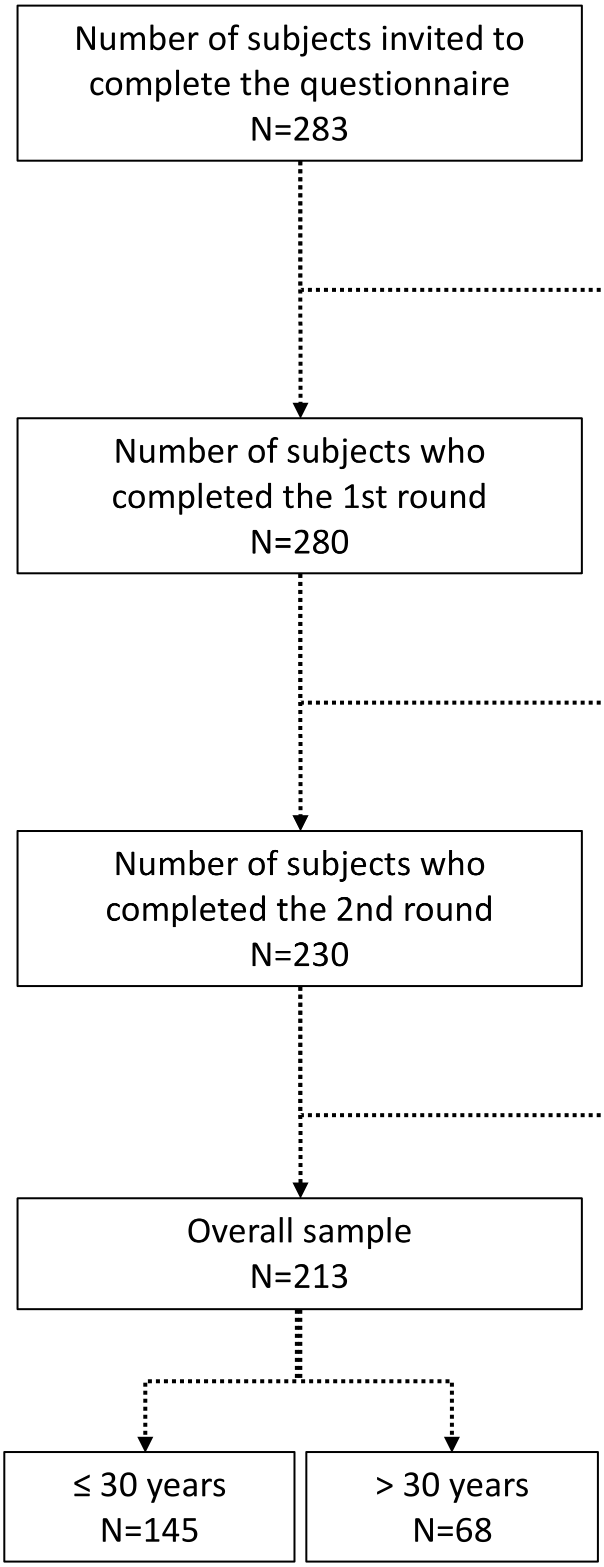

Figure S1. Flowchart of the inclusion criteria of study participants.

Table S1. Kappa correlation coefficients (weighted and non-weighted) and corresponding 95% confidence intervals (95%CI) to assess test-retest reliability of dietary behaviours between the two time points in pilot validation phase.

Food Items	Kappa (95%CI)	N	Kappa (95%CI)	N
**Other variables**				
Water consumption^	0.82 (0.74;0.90)	144	0.66 (0.49;0.82)	68
Alcohol consumption outside meals	0.54 (0.38;0.70)	107	0.53 (0.25;0.81)	46
Fresh white meat	0.66 (0.52;0.79)	128	0.60 (0.40;0.80)	60
Fresh red meat	0.65 (0.52;0.79)	121	0.51 (0.29;0.74)	53
Non-refined cereals	0.62 (0.43;0.81)	63	0.58 (0.27;0.90)	25
Doing breakfast	0.81 (0.66;0.96)	144	1.00 (1.00;1.00)	67
Homemade sweets	0.51 (0.33;0.68)	131	0.60 (0.32;0.89)	54
Length of meals^	0.58 (0.46;0.69)	145	0.70 (0.56;0.85)	68
Use of herbs instead of salt	0.71 (0.59;0.83)	144	0.76 (0.55;0.96)	68
Frequency of consumption of non-refined cereals	0.55 (0.40;0.70)	139	0.72 (0.52;0.91)	65
Eating outside meals	0.62 (0.50;0.75)	145	0.78 (0.63;0.93)	68
Olive oil consumption^	0.64 (0.53;0.76)	144	0.31 (0.02;0.60)	68
Skimmed daily products	0.67 (0.51;0.82)	143	0.54 (0.31;0.77)	68
Seasonal fuits	0.43 (0.25;0.62)	114	0.53 (0.24;0.82)	52
Seasonal vegetables	0.49 (0.31;0.66)	140	0.40 (0.13;0.67)	65
Changes in usual diet	0.36 (0.15;0.57)	139	0.27 (-0.04;0.57)	66
Frequency of eating out	0.52 (0.42;0.63)	145	0.61 (0.52;0.71)	65

^weighted Kappa
